# Safety and efficacy of short-term tirofiban combined with dual antiplatelet therapy after flow diverter placement for intracranial aneurysms: a multicenter retrospective study and nomogram for thromboembolic event prediction

**DOI:** 10.3389/fneur.2025.1689308

**Published:** 2025-11-19

**Authors:** Xiaoning Liu, Yunpeng Lin, Bingcheng Ren, Yang Li, Jiwen Wang, Xiangbo Liu, Yidi Wang, Chenguang Guo, Fushun Xiao, Shiqing Mu

**Affiliations:** 1 Department of Neurosurgery, Tianjin Medical University General Hospital, Tianjin, China; 2 Nankai District Center for Disease Control and Prevention, Tianjin, China; 3 Department of Interventional Neuroradiology, Beijing Neurosurgical Institute, Beijing Tiantan Hospital, Capital Medical University, Beijing, China

**Keywords:** aneurysm, flow diversion, thromboembolic events, predictive models, multicentric, retrospective study

## Abstract

**Background:**

Flow diverters (FDs) are increasingly applied for intracranial aneurysms, but their high metal coverage raises thromboembolic risk. Dual antiplatelet therapy (DAPT) with aspirin and clopidogrel is standard, yet clopidogrel resistance, especially in Asian populations, reduces efficacy. Tirofiban, a glycoprotein IIb/IIIa inhibitor, may enhance perioperative protection. This study evaluated the safety and efficacy of adding short-term tirofiban to DAPT after FDs treatment and developed a model to predict thromboembolic events (TEEs).

**Methods:**

We retrospectively analyzed 319 patients with unruptured aneurysms treated with FDs across multiple centers (2018–2022). Patients received either DAPT alone (group 1) or DAPT plus tirofiban (group 2). After propensity score matching (140 per group), ischemic and hemorrhagic complications were compared. Predictive factors for TEEs were identified using Lasso-logistic regression, and a nomogram was constructed.

**Results:**

A total of 389 aneurysms in 319 patients were included in the statistical analysis. There were no statistically significant differences in the baseline characteristics of the patients and aneurysms between the groups, indicating comparability. After PSM, 140 patients were included in each group for comparison. The group 1 vs. the group 2 (Early postoperative complications): TEEs (3.6% vs. 5.0%, *p* = 0.768), intracranial hemorrhage (0% vs. 0.7%, *p* = 0.390); The group 1 vs. the group 2 (Long-term postoperative complications): TEEs (3.3% vs. 5.7%, *p* = 0.370), intracranial hemorrhage (3.6% vs. 1.4%, *p* = 0.444), and peripheral bleeding events (2.9% vs. 6.5%, *p* = 0.256) showed no statistically significant differences. Multivariable logistic regression identified maximum aneurysm diameter (OR = 1.153, 95% CI: 1.087–1.223, *p* < 0.0001) as significant risk factor for TEEs, while diameter of the feeding artery (OR = 0.442, 95% CI: 0.286–0.682, *p* = 0.0003) was protective factor. A nomogram based on these factors achieved a C-index of 0.723.

**Conclusion:**

Tirofiban combined with DAPT in flow diverter treatment for intracranial aneurysms demonstrated good safety without increasing bleeding risk, though its efficacy advantage over DAPT alone was not evident. The proposed nomogram enables individualized TEE risk prediction and supports personalized antiplatelet management.

## Introduction

In recent years, with the rapid advancement of neurointerventional techniques, flow diverters (FDs) have gradually emerged as the primary treatment option for complex intracranial aneurysms. Although conventional coil embolization and stent-assisted coil embolization can achieve satisfactory occlusion in certain cases, their efficacy remains limited for large, giant, or wide-neck aneurysms, which are more susceptible to incomplete occlusion and high recurrence rates (1–3). FDs function by deploying a braided stent with high metal coverage within the parent artery, thereby reconstructing the hemodynamics at the aneurysm neck, reducing intra-aneurysmal blood flow, and promoting thrombus formation and endothelialization, ultimately leading to durable aneurysm occlusion (4, 5). However, FDs have relatively higher porosity and pore density, which increase the likelihood of platelet adhesion and activation. Consequently, thrombus formation has become one of the most frequent and potentially life-threatening perioperative complications associated with FDs treatment (6–8). Striking a balance between effective aneurysm healing and the minimization of thromboembolic events remains a key challenge and focus of ongoing clinical practice and research.

To date, the combination of aspirin and clopidogrel remains the most widely used regimen for the prevention of thromboembolic events (9). However, compared with other antiplatelet agents, clopidogrel resistance represents a major limitation to its effectiveness, with reported resistance rates ranging from 4 to 30%, and even up to 70% in certain Asian populations (10). Glycoprotein IIb/IIIa inhibitors, such as tirofiban, are commonly employed to prevent ischemic events, particularly for the prevention and emergency management of thromboembolic complications (11). Agents in this class—including tirofiban, abciximab, and eptifibatide—are increasingly utilized in neurointerventional procedures. Tirofiban exhibits a rapid and reversible pharmacodynamic profile with a short plasma half-life of approximately 2 to 2.5 h. It acts by blocking platelet surface Gp IIb/IIIa receptors, thereby inhibiting fibrinogen binding, platelet aggregation, and subsequent thrombus formation (12).

This study aims to investigate strategies for optimizing postoperative antiplatelet therapy, with the goal of developing more evidence-based and individualized regimens to prevent postoperative complications. Meanwhile, the study evaluated the efficacy and safety of a triple antiplatelet regimen consisting of aspirin, clopidogrel, and tirofiban following FDs procedures. Furthermore, a predictive model for ischemic events was developed using Lasso-logistic regression, and a corresponding nomogram was established to assist clinicians in tailoring individualized treatment strategies for patients.

## Methods

### Patient selection

This study was a retrospective analysis utilizing data from a neuroendovascular database jointly maintained by two neurointerventional centers. Patients were eligible for inclusion if they had an unruptured intracranial aneurysm treated with a FD and received combined therapy with aspirin, clopidogrel, and tirofiban between June 2018 and September 2022. Baseline demographic information, aneurysm characteristics (including size, morphology, and location), procedural details, and clinical outcomes were systematically collected. All analyses were performed retrospectively using anonymized clinical data. The study protocol was reviewed and approved by the Institutional Review Board (Approval No.: IRB2024-YX-296-01). Owing to its retrospective design, the requirement for informed consent was waived.

All procedures were performed under general anesthesia, and all patients underwent cerebral digital subtraction angiography (DSA), including three-dimensional rotational angiography, to assess aneurysm size, shape, and location, thereby assisting in the proper selection of FDs device size and deployment. The Pipeline Embolization Device (PED; Medtronic Neurovascular, Irvine, California, USA) or Tubridge (MicroPort NeuroScientific Corporation, Shanghai, China) was used in all procedures.

### Antiplatelet treatment and anticoagulation

All aneurysm procedures were conducted under general anesthesia with systemic heparinization. A total of 319 patients received dual antiplatelet therapy (aspirin 100 mg daily and clopidogrel 75 mg daily) for 3 to 5 days before undergoing the endovascular procedure. Preoperative platelet function testing was performed using adenosine diphosphate (ADP)-induced aggregation assays, arachidonic acid–induced assays, or thromboelastography (TEG); ADP resistance (ADR) was defined as >50%. During the procedure, all patients received systemic heparinization with an initial bolus of 30–40 U/kg administered before stent deployment, followed by supplemental doses of 1,000 U every hour thereafter. Postoperatively, dual antiplatelet therapy (DAPT) was continued for 6 months, after which single antiplatelet therapy (SAPT) with aspirin was maintained for an additional year. During the procedure, all patients received systemic heparinization with an initial bolus of 30–40 U/kg administered before stent deployment, followed by supplemental doses of 1,000 U every hour thereafter.

Tirofiban was administered intraoperatively when thrombus formation or suspected thrombus formation was observed, when slow or stagnant flow occurred within the stent, when incomplete stent apposition was noted, or when the operator judged a high risk of thromboembolic events based on angiographic findings. Under these circumstances, patients received an intravenous infusion of tirofiban (50 μg/mL) at a maintenance rate of 0.1 μg/kg/min (approximately 4–6 mL/h for a 70 kg patient) for 12 h during the procedure, in combination with the aforementioned medications.

### Outcome and follow up

The primary outcomes were the occurrence of TEEs and hemorrhagic events (categorized as intracranial and peripheral) in the early (within 1 week) and late (follow-up) phases. TEEs include all types of asymptomatic TEEs (thrombus formation during endovascular procedures, asymptomatic stent occlusion), transient ischemic attack (TIA), or permanent ischemic stroke. Major hemorrhage is defined as any symptomatic intracranial hemorrhage or any peripheral bleeding that requires intensive care or involves life-threatening bleeding requiring modification of SAPT. Peripheral vascular bleeding is defined as bleeding located in the limbs (upper or lower) or other regions distant from major central blood vessels, including subcutaneous hemorrhage, gingival bleeding, epistaxis, and retinal hemorrhage. Patients underwent clinical and angiographic follow-up at 6 months after the procedure to evaluate aneurysm occlusion. Long-term clinical follow-up, including the modified Rankin Scale (mRS), was performed at 12 months during outpatient visits to assess functional outcomes.

### Statistical analysis

Data analysis was performed using the R Studio statistical software package (version 4.4.2). Missing values were imputed using multiple imputation methods. Comparisons between categorical variables were made using the χ2 test or Fisher’s exact test, while continuous variables were analyzed using the unpaired t-test or Mann–Whitney U test. A *p*-value of <0.05 was considered statistically significant. PSM was used to adjust for potential differences between the two groups in variables such as AA (arachidonic acid), ADP, age, sex, smoking, alcohol, hypertension, diabetes, red blood cell count, platelet count, hemoglobin levels, aneurysm maximum diameter, and neck size. Matching was performed using a 1:1 nearest neighbor matching method based on propensity score logit, with a caliper set at 0.2 standard deviations and without replacement. To address the issue of class imbalance between patients with and without ischemic events, the Synthetic Minority Over-sampling Technique (SMOTE) was applied to the dataset prior to model construction (13).

Furthermore, in this study, the dataset was randomly divided into training and test sets in a 6:4 ratio. Least absolute shrinkage and selection operator (Lasso) regression was employed to identify potential risk factors, and a predictive model was subsequently developed using logistic regression, incorporating the variables selected by Lasso. Odds ratios (OR) and their corresponding 95% confidence intervals (CI) were calculated through logistic regression analysis. The optimal *λ* value was determined via cross-validation, and the model’s predictive performance was evaluated using the concordance index (C-index) and receiver operating characteristic (ROC) curves. Model discrimination and calibration were further assessed using calibration plots. In addition, decision curve analysis (DCA) was conducted to evaluate the clinical utility of the model. Finally, the results were visualized in the form of a nomogram to facilitate clinical interpretation.

## Results

### Baseline characteristics of patients and aneurysms

A total of 319 patients with unruptured intracranial aneurysms who underwent FDs treatment were included in this study. All patients received treatment with aspirin and clopidogrel, and 169 patients were additionally treated with tirofiban postoperatively. PSM was performed in this study, resulting in 140 matched pairs of patients. Table 1 demonstrates the comparability of baseline characteristics between the two groups after PSM matching. Supplementary Table S1 shows the baseline characteristics of the two groups.

**Table 1:** Angiography and clinical features.

Propensity score matched for the treatment of aneurysms (*n* = 280 patients)			
Variables	DAPT	DAPT and tirofiban	*P*-value
	(*N* = 140)	(*N* = 140)	
Sex			
Male	54 (38.6%)	47 (33.6%)	>0.999
Female	86 (61.4%)	93 (66.4%)	
Age			
Mean (SD)	55.4 (10.9)	55.9 (11.4)	0.477
Alcohol	19 (13.6%)	18 (12.9%)	>0.999
Smoking	27 (19.3%)	23 (16.4%)	0.640
Hypertension	66 (47.1%)	69 (49.3%)	0.811
Diabetes	10 (7.2%)	13 (9.4%)	0.808
Symptom	91 (65.0%)	85 (60.7%)	0.536
Dizziness	41 (29.3%)	37 (26.4%)	0.689
Headache	40 (28.6%)	33 (23.6%)	0.414
Cranial nerve paralysis	9 (6.4%)	8 (5.7%)	>0.999
History of stroke	0 (0%)	3 (2.1%)	0.246
AA	9.70 (8.96)	9.77 (7.10)	0.613
ADP	36.6 (16.0)	37.6 (14.6)	0.805
ADR	30 (21.4%)	26 (18.6%)	0.654
Maximum diameter			
Mean (SD)	8.92 (6.95)	8.38 (6.05)	0.767
Width			
Mean (SD)	6.90 (5.00)	6.31 (4.69)	0.272
Neck width			
Mean (SD)	6.51 (4.79)	6.31 (4.69)	0.227
Diameter of the feeding artery			
Mean (SD)	3.71 (1.11)	3.71 (1.47)	0.699
Aneurysm location			
Anterior circulation	122 (87.1%)	115 (82.1%)	0.220
Posterior circulation	18 (12.9%)	25 (17.9%)	
Admission_mRS			
0–2	138 (98.6%)	135 (96.4%)	0.450
>2	2 (1.4%)	5 (3.6%)	
Intraoperatively			
Thrombosis	1 (0.7%)	0 (0%)	0.390
Other complications	1 (0.7%)	3 (2.2%)	0.310
Postoperatively			
Ischemic complication	5 (3.6%)	7 (5.0%)	0.768
Cerebral hemorrhage	0 (0%)	1 (0.7%)	0.390
Other complications	0 (0%)	6 (4.3%)	0.037
Death	0 (0%)	1 (0.7%)	0.390
mRS score(3 Day)			
0–2	133 (95.0%)	138 (98.6%)	0.170
>2	7 (5.0%)	2 (1.4%)	
mRS score(discharge)			
0–2	132 (94.3%)	137 (97.9%)	0.210
>2	8 (5.7%)	3 (2.1%)	
Follow-up			
Follow up time			
Mean (SD)	9.51 (3.76)	9.92 (3.63)	0.299
Ischemic complication	4 (3.3%)	8 (5.7%)	0.370
Hemorrhagic complication	5 (3.6%)	2 (1.4%)	0.444
Peripheral hemorrhagic	4 (2.9%)	9 (6.5%)	0.256
mRS score			
0–2	134 (95.7%)	137 (97.9%)	0.500
>2	6 (4.3%)	3 (2.1%)	

Tips: the full table is available in the Supplementary material.

### Procedure and outcomes

In this study, propensity score matching (PSM) was performed, as shown in Table 1, to minimize selection bias and control for potential confounding variables, including AA and ADP–induced platelet function, age, sex, smoking status, alcohol consumption, hypertension, diabetes, red blood cell count, platelet count, hemoglobin level, aneurysm maximum diameter, and neck size. Early postoperative complications: ischemic complications (5/139, 3.6% vs. 7/139, 5.0%, *p* = 0.768); cerebral hemorrhage (0/139, 0% vs. 1/139, 0.7%, *p* = 0.390); other complications (0/139, 0% vs. 6/139, 4.3%, *p* = 0.037); death (0/139, 0% vs. 1/139, 0.7%, *p* = 0.390). Long-term follow-up: the mean follow-up duration was 9.51 ± 3.76 months in group 1 and 9.92 ± 3.63 months in group 2 (*p* = 0.299). Ischemic complications occurred in 4/139 (3.3%) vs. 8/139 (5.7%) (*p* = 0.370); hemorrhagic complications in 5/139 (3.6%) vs. 2/139 (1.4%) (*p* = 0.444); and peripheral hemorrhagic events in 4/139 (2.9%) vs. 9/139 (6.5%) (*p* = 0.256). No statistically significant differences were observed between the two groups.

Among the 319 patients in Supplementary Table S1, a total of 5 patients (5/150, 3.3%) in group 1 experienced postoperative TEEs. One patient (1/150, 0.7%) underwent re-release due to poor opening of the FD stent, followed by TEEs. One patient (1/150, 0.7%) developed in-stent thrombosis during the procedure. One patient (1/150, 0.7%) experienced aneurysm rupture during the procedure. One patient (1/150, 0.7%) had an allergic reaction. In group 2, a total of 8 patients (8/169, 4.7%) experienced postoperative TEEs. Among them, 2 patients (2/169, 1.2%) had poor stent opening, and one required the placement of a second stent, with both patients developing TEEs postoperatively. One patient (1/169, 0.6%) experienced early TIA (transient vision loss in the left eye). One patient (1/169, 0.6%) developed intracranial hemorrhage postoperatively, leading to death due to hemorrhage extending into the ventricles. Two patients (2/169, 1.2%) had allergic reactions, and one patient (1/169, 0.6%) with a gastric duodenal adenoma experienced gastrointestinal discomfort. During the procedure, 2 patients (2/169, 1.2%) had branch artery occlusion.

In long-term follow-up, there was no statistically significant difference between group 1 and group 2 (9.35 ± 3.65 months vs. 9.79 ± 3.61 months, *p* = 0.234). TEEs: group 1 (4/150, 2.7%) vs. group 2 (13/169, 7.7%), *p* = 0.081; Intracranial hemorrhage events: group 1 (4/150, 2.7%) vs. group 2 (3/169, 1.8%), *p* = 0.873. In group 1, 4 patients (4/150, 2.7%) and in group 2, 2 patients (2/169, 1.2%) developed intracranial hemorrhage after discharge. Peripheral bleeding events: group 1 (4/150, 2.7%) vs. group 2 (12/169, 7.1%), *p* = 0.12. In group 1, there were 3 cases of skin bruising and 1 case of retinal hemorrhage. In group 2, there were 9 cases (5.33%) of skin bruising, 2 cases (1.18%) of gingival bleeding, and 1 case (0.59%) of increased menstrual bleeding.

### Lasso regression and log model development

**Figure 1:**
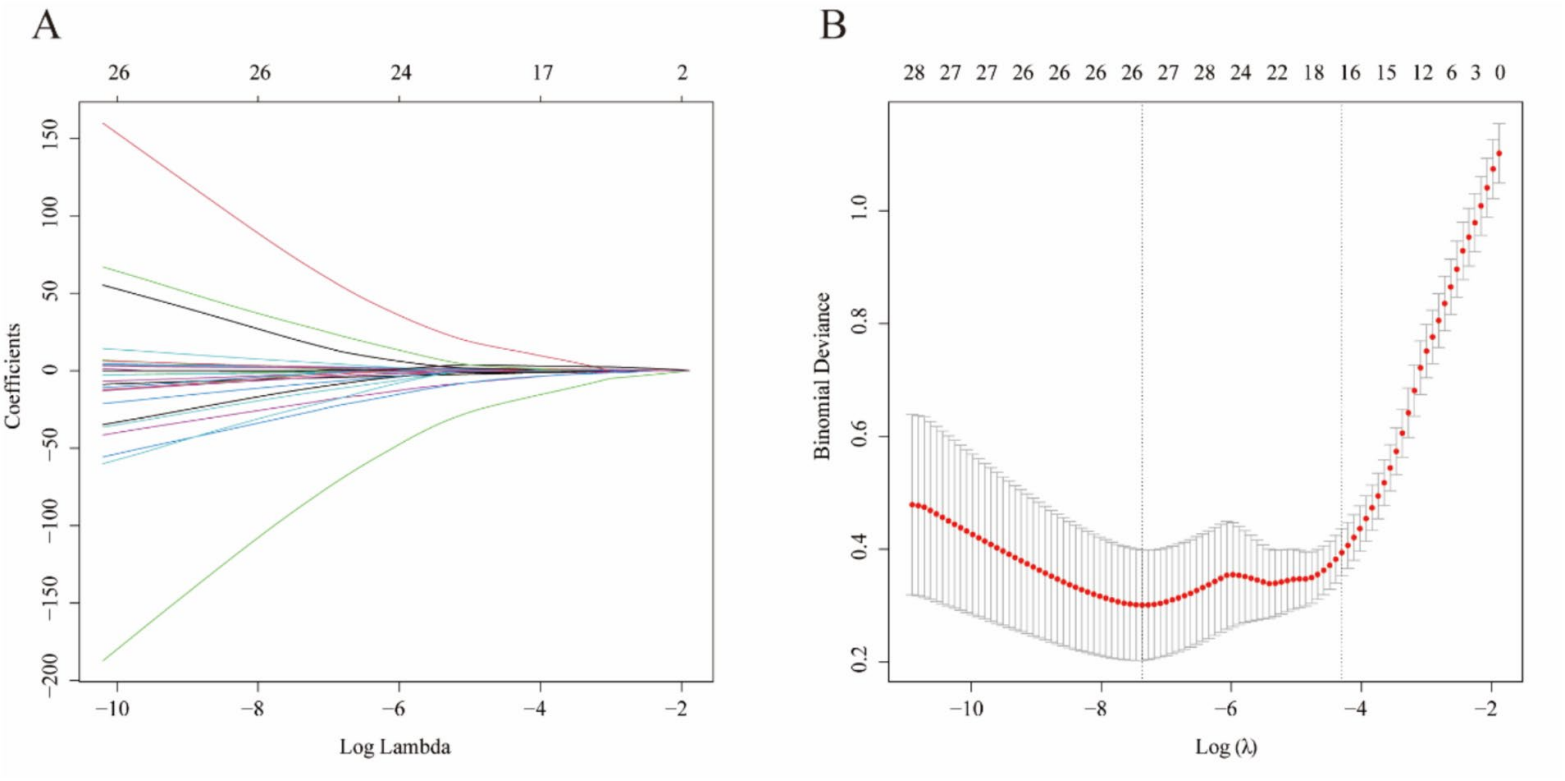
Lasso regression-based variable selection. **(A)** The trajectory of each variable coefficient as a function of log(*λ*). Each colored line represents the coefficient path of a variable during LASSO regularization. **(B)** The optimal value of the regularization parameter λ was determined by 10-fold cross-validation, where the red dots represent the mean binomial deviance and the error bars indicate standard deviations. The vertical dotted lines correspond to the λ values with minimum deviance and one standard error from the minimum, respectively.

Lasso regression was used to select variables from the dataset, with the coefficient variation characteristics of these variables shown in Figure 1A. A 20-fold cross-validation was performed for iterative analysis, as shown in Figure 1B. The final variables retained included the maximum aneurysm diameter and the diameter of the feeding artery. Based on the parameters selected by Lasso regression, a logistic regression model was further developed (Table 2), yielding an area under the ROC curve (AUC) of 0.723 (95% CI: 0.606–0.840).

**Table 2:** Multivariable logistic regression to identify factors associated with TEEs.

Variable	OR (95% CI)	*P*-value
Maximum diameter	1.153 (1.087–1.223)	<0.0001
Diameter of the feeding artery	0.442 (0.286–0.682)	0.0003

Tips: The full table is available in the Supplementary material.

### Nomograms and forest charts as tools for visualization

**Figure 2:**
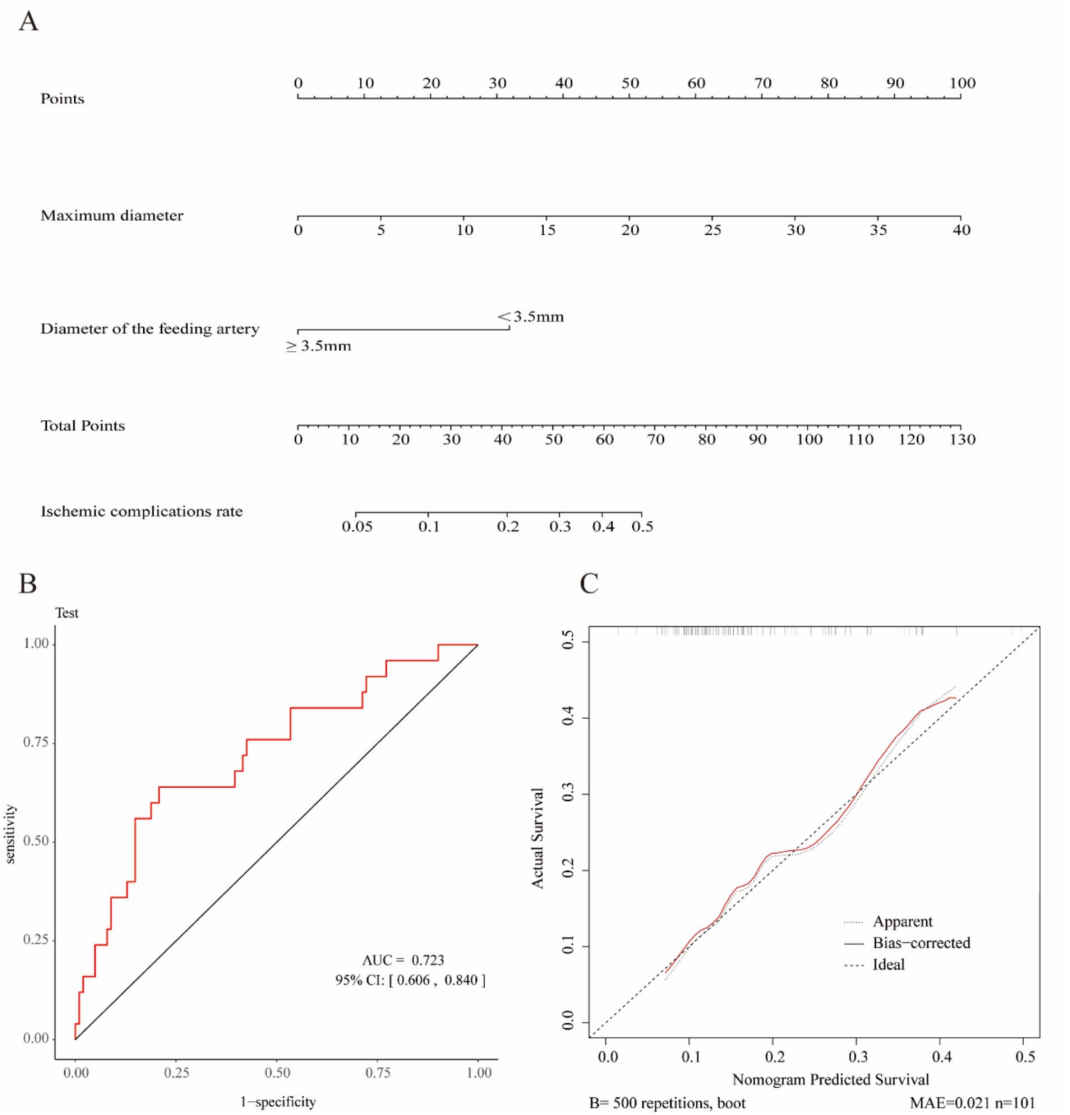
Construction and validation of the nomogram for predicting thromboembolic events (TEEs) after flow diverter treatment in patients with intracranial aneurysms. **(A)** Nomogram developed to estimate the probability of thromboembolic events based on independent predictors identified by LASSO logistic regression. **(B)** Receiver operating characteristic (ROC) curve of the nomogram, showing an area under the curve (AUC) of 0.723 (95% CI: 0.606–0.840), indicating acceptable discriminative performance. **(C)** Calibration curve of the nomogram generated by bootstrap resampling, demonstrating good agreement between predicted and observed probabilities.

**Figure 3:**
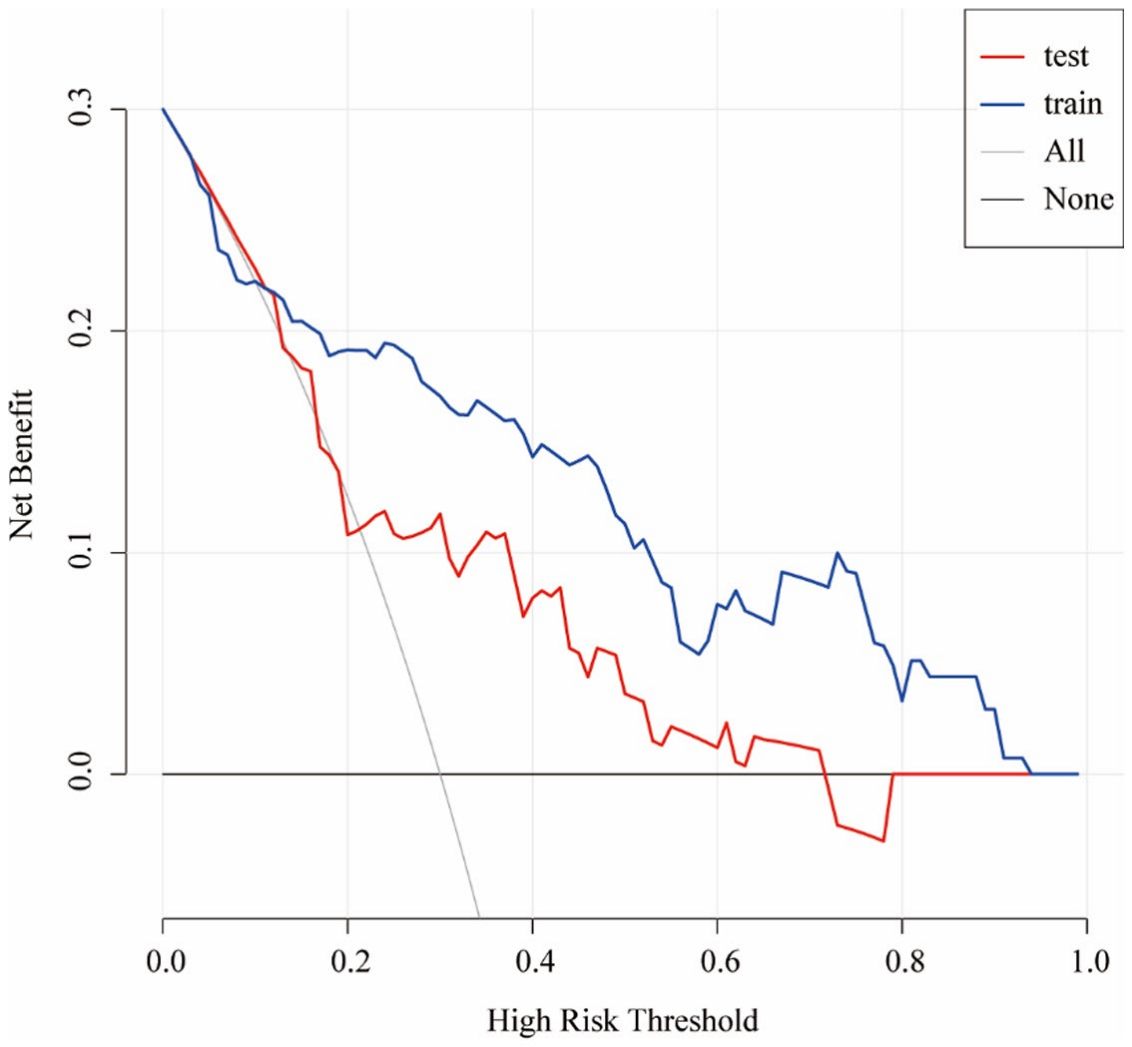
Decision curve analysis (DCA) of the nomogram for predicting TEEs after flow diverter treatment. The decision curve demonstrates the clinical net benefit of the nomogram at different threshold probabilities in both the training (blue line) and test (red line) cohorts. The gray line (“All”) represents the assumption that all patients would experience TEEs, whereas the black line (“None”) assumes that no patient would experience TEEs. The results indicate that the nomogram provides greater clinical benefit than the treat-all or treat-none strategies across a wide range of threshold probabilities.

**Figure 4:**
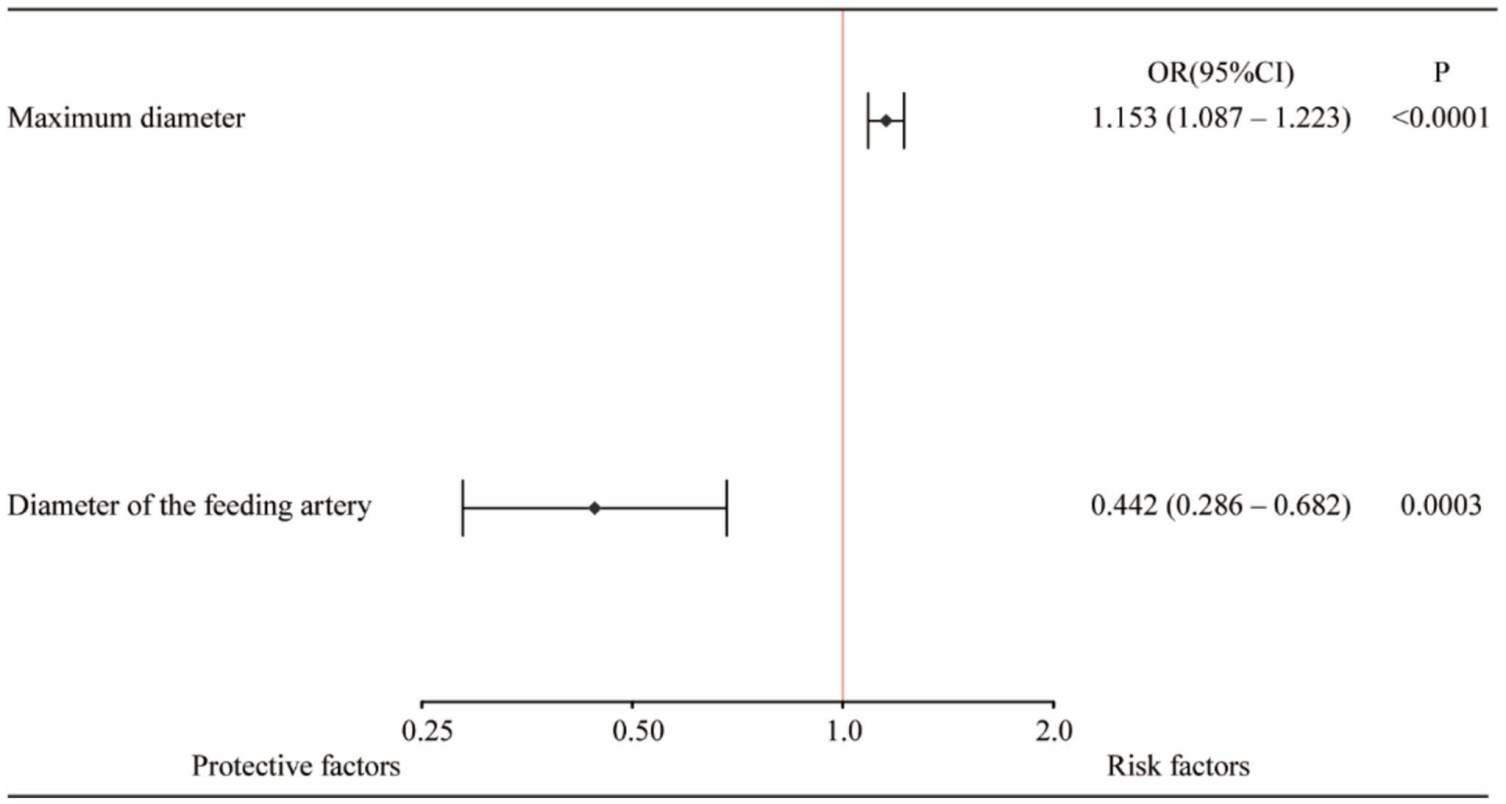
Forest plot of independent predictors of thromboembolic events (TEEs) after flow diverter treatment in patients with intracranial aneurysms. Multivariate logistic regression analysis identified maximum aneurysm diameter and feeding artery diameter as independent predictors of TEEs. A larger aneurysm diameter was associated with an increased risk of thromboembolic events (OR = 1.153, 95% CI: 1.087–1.223, *p* < 0.0001), whereas a larger feeding artery diameter (≥ 3.5 mm) was a protective factor (OR = 0.442, 95% CI: 0.286–0.682, *p* = 0.0003).

In summary, the Lasso-logistic regression model was developed to predict the risk of TEEs in patients with aneurysms treated using FDs. To enhance clinical applicability, the complex mathematical model was transformed into a nomogram, and its performance was evaluated using receiver operating characteristic (ROC) curves and calibration plots (Figure 2). The clinical utility of the model was further evaluated through DCA, as illustrated in Figure 3. The results demonstrated that, within the probability range of approximately 0.1–0.6, both the training set (blue line) and the test set (red line) showed greater net benefit compared with the “All” and “None” strategies. The final model incorporated the following independent predictors: maximum aneurysm diameter (OR = 1.153, 95% CI: 1.087–1.223, *p* < 0.0001) and diameter of the feeding artery (OR = 0.442, 95% CI: 0.286–0.682, *p* = 0.0003). These results were further visualized in the form of a dendrogram, as shown in Figure 4. The maximum aneurysm diameter was identified as a risk factor for TEEs, whereas a larger feeding artery diameter served as a protective factor. In the nomogram, the scores assigned to each variable can be summed, and the total score corresponds to the predicted probability of TEEs occurrence.

## Discussion

In our study, after PSM, the incidence of early postoperative ischemic complications in the tirofiban group was 5.0%, while the hemorrhagic complication rate was 0.7%. The long-term ischemic and hemorrhagic complication rates were 5.7 and 1.4%, respectively. No significant differences were observed between groups, suggesting that the addition of tirofiban to DAPT following FDs treatment for intracranial aneurysms appears to be safe; however, its therapeutic efficacy warrants further investigation. Furthermore, multivariable analysis identified alcohol consumption and larger aneurysm diameter as independent risk factors for postoperative TEEs, whereas a greater feeding artery diameter was found to be a protective factor—indicating that larger vessel caliber was associated with a reduced likelihood of TEEs. Based on these findings, we developed a predictive model to estimate the risk of long-term postoperative ischemic events in patients with intracranial aneurysms and constructed a corresponding nomogram to facilitate individualized risk assessment.

Previous studies have confirmed that tirofiban is effective both in preventing thromboembolic events and in managing acute thrombotic complications (11, 14). In certain patients who develop acute thrombosis during the procedure, tirofiban is administered through intra-arterial or intravenous injection, and postoperative oral DAPT is typically withheld temporarily. Once the patient’s condition stabilizes, DAPT is resumed; however, during this transition period, the continuity of the antiplatelet effect may be compromised. In contrast, the concurrent use of DAPT and tirofiban maintains continuous antiplatelet coverage, obviates the need for bridging therapy, and effectively reduces the risk of thrombus formation. Furthermore, existing evidence indicates that this combination does not increase the incidence of bleeding or other adverse events, supporting the safety of tirofiban administration in conjunction with DAPT for patients with unruptured intracranial aneurysms undergoing FDs treatment (15, 16).

Globally, antiplatelet therapy strategies following flow diversion vary widely, and a universally accepted standard has yet to be established. Findings from the SAFEST study indicated that 67% of physicians preferred the combination of low-dose aspirin and clopidogrel. With increasing clinical experience, the use of alternative P2Y12 receptor inhibitors has become more common, with ticagrelor used in 21% of cases and prasugrel in 10%. Ticagrelor is generally preferred due to its greater availability and lower associated bleeding risk. Management strategies for P2Y12 inhibitor resistance primarily include dose escalation (17%) or switching to an alternative antiplatelet agent (83%) (17). The antiplatelet regimen may represent a potential source of residual confounding. However, there is currently no consensus on the optimal duration or regimen of antiplatelet therapy after flow diversion.

In most studies on tirofiban, the method of administration, dosage, and dosing regimen have not yet established a universally accepted safety standard. In a study by Nohra Chalouhi, 16 patients out of 67 (30 of whom had SAH) received a bolus injection of tirofiban at a dose of 0.4 μg/kg/min for 30 min, followed by a maintenance dose of 0.1 μg/kg/min for 21 min. The incidence of intracranial hemorrhagic complications reached as high as 18.8%. Subsequently, 51 patients switched to a continuous infusion of 0.1 μg/kg/min of tirofiban, reducing the incidence of intracranial hemorrhage to 1.9% (18). It is worth noting that the unruptured aneurysm patients in this study did not undergo pre-treatment with DAPT (19). Additionally, in a study by Edgar A. Samaniego and colleagues, tirofiban at a dose of 0.10 μg/kg/min was administered during the procedure (either before or after stent deployment) on top of DAPT. Among 141 aneurysm patients (110 with SAH), 5 patients (3.4%) had symptomatic brain hemorrhages (20). The main limitations of these two studies are that only a subset of patients underwent FDs treatment, and the proportion of patients with ruptured aneurysms was not negligible. In cases of aneurysm rupture, platelet activation, a pronounced elevation in inflammatory markers, and the occurrence of acute infarct events detectable by MRI have been well documented (21). In the present study, ruptured aneurysms were rigorously excluded to eliminate the potential confounding effects of aneurysm status on prognostic evaluation. This carefully controlled study subsequently validated the clinical efficacy and safety of tirofiban in combination with DAPT among patients with unruptured aneurysms, providing high-quality evidence to support the optimization of postoperative antiplatelet therapy strategies in this population.

It is noteworthy that a total of three patients across both groups developed TEEs postoperatively, primarily due to incomplete stent expansion. Similarly, improper stent positioning during FDs deployment, followed by retrieval and re-deployment, likely resulted in endothelial injury and subsequent small-branch artery occlusion—factors that may have contributed to the elevated risk of TEEs. In the present study, the incidence of TEEs was comparable between the two groups and remained at a relatively low level, suggesting that the addition of tirofiban to DAPT did not confer a significant advantage in preventing TEEs, although the overall safety profile was acceptable. Nevertheless, the tirofiban group exhibited a slightly higher rate of TEEs during both the postoperative and follow-up periods compared with the DAPT-only group.

A previous meta-analysis demonstrated that FDs treatment for intracranial aneurysms is associated with a higher incidence of TEEs in the posterior circulation compared with the anterior circulation (10) (12.9% vs. 17.9%), and a greater proportion of FDs were deployed in the posterior circulation in the tirofiban group. Although no statistically significant difference in long-term TEEs was observed between the two groups, the absolute difference (2.7% vs. 7.7%) remains clinically relevant. This lack of significance may be attributed to the limited sample size and insufficient statistical power rather than the absence of a genuine effect. Accordingly, these findings should be interpreted with caution, and larger prospective or multicenter randomized studies are needed to validate this potential association.

Additionally, one representative case involved a 51-year-old male with a basilar artery aneurysm measuring 25 × 22 mm with a 16 mm neck and a history of subarachnoid hemorrhage previously treated by aneurysm embolization. The patient underwent FDs stent placement with a baseline mRS score of 5. No ischemic or hemorrhagic complications occurred postoperatively, and he was discharged with an unchanged mRS score of 5. However, he died of a pulmonary infection six months later. Notably, tirofiban was used as a rescue therapy for intraoperative complications, which may have contributed to the higher incidence of TEEs observed in the tirofiban group.

No significant differences in bleeding events, including peripheral vascular hemorrhage, were observed between the two groups, confirming that the addition of tirofiban did not increase the risk of bleeding and further supporting the safety of the DAPT plus tirofiban regimen. However, two patients in the tirofiban group developed intracranial hemorrhage after discharge (mRS = 2). Previous studies have indicated that FDs procedures may introduce unique risks not typically seen with conventional endovascular techniques, such as distal intraparenchymal hemorrhage (DIPH). The markedly increased flow velocity in arteries associated with DIPH and the resultant imbalance in distal perfusion may play a crucial role in its development. Although such events are rare, they warrant clinical awareness and careful monitoring (22, 23).

In this study, a predictive model for postoperative ischemic complications was developed using Lasso-logistic regression within a retrospective design. The resulting nomogram identified maximum aneurysm diameter and feeding artery diameter as independent predictors of TEEs. Previous studies have extensively investigated risk factors associated with complications following endovascular treatment (24). Aneurysm size is an important predictor of ischemic complications; larger aneurysms are associated with an increased risk of ischemic events and subsequent clinical complications. In addition, large aneurysms may compress adjacent neural structures, leading to neurological deficits (25). Previous studies have confirmed that advanced age, alcohol consumption, a history of hypertension, and elevated total cholesterol levels are associated with an increased risk of ischemic events (26–28). The diameter of the feeding artery was identified as a protective factor, which may be explained by two potential mechanisms: First, from a pathophysiological standpoint, atherosclerotic changes cause endothelial thickening and luminal narrowing, thereby reducing the arterial diameter. Second, as the feeding artery becomes narrower, blood flow velocity increases; elevated shear stress may cause flow disturbances and turbulence, which can further aggravate endothelial dysfunction. Moreover, the deployment of a FD may exacerbate endothelial injury, further promoting platelet activation and increasing the risk of thrombus formation.

Based on the Lasso-logistic regression analysis, we identified independent predictors and constructed a nomogram to estimate the probability of postoperative ischemic complications. Each variable corresponds to a specific value on the “Points” scale, and the sum of the scores from the two variables yields the “Total Points.” The predicted probability of postoperative ischemic events can then be determined according to the scale at the bottom of the nomogram. A larger aneurysm diameter corresponds to a higher total score, indicating an increased risk of ischemic complications, whereas a feeding artery diameter of ≥3.5 mm is associated with a lower total score and consequently a reduced risk. This nomogram provides an intuitive and practical tool for individualized risk assessment and clinical decision-making regarding postoperative ischemic complications. The clinical significance of this predictive model lies in its capacity for personalized analysis and patient-specific risk assessment. The nomogram enables clinicians to rapidly quantify the risk of TEE occurrence based on individual patient characteristics, thereby facilitating personalized treatment planning and effective risk communication. For high-risk patients, more aggressive preventive or therapeutic strategies may be considered.

For patients exhibiting clopidogrel resistance or borderline responsiveness, we did not substitute ticagrelor or other P2Y12 receptor antagonists. Throughout the study period, aspirin combined with clopidogrel remained the standard antiplatelet regimen for neurointerventional procedures in our institution. For patients with borderline or mild ADP resistance, ticagrelor was not substituted; instead, tirofiban was administered intraoperatively or postoperatively as a bridging therapy when clinically indicated. The results demonstrated that the addition of tirofiban did not increase the risk of bleeding, and the incidence of TEEs did not differ significantly from that observed with DAPT alone. However, we acknowledge that the outcomes may differ in patients with high-grade clopidogrel resistance, and large-scale prospective studies are warranted to further validate these findings. In certain emergency neurointerventional scenarios—such as posterior communicating artery aneurysms presenting with acute neurological compression symptoms—patients may be unable to undergo standard preoperative preparation. In such situations, the combination of DAPT with tirofiban may represent a safe and feasible antiplatelet strategy; however, its specific clinical utility requires further validation through additional studies.

This study has several limitations. First, as a retrospective observational study, it is subject to inherent biases; however, PSM was applied to minimize the potential influence of confounding variables during data analysis. Second, although the study was conducted across multiple centers, inter-center heterogeneity in equipment, procedural techniques, and operator experience may have introduced confounding effects on the outcomes. Third, this study focused exclusively on patients treated with FDs; stent-assisted coiling (SAC), another widely used treatment modality, was not included in the analysis. Finally, the overall sample size was relatively limited, which may have affected the statistical power and introduced potential bias into the findings.

## Conclusion

In the treatment of intracranial aneurysms with FDs, the addition of the glycoprotein IIb/IIIa inhibitor tirofiban to DAPT did not show a significant advantage in preventing postoperative TEEs, but demonstrated good overall safety. The use of tirofiban did not increase the incidence of bleeding events. The proposed nomogram can be applied for individualized assessment of TEEs risk, thereby supporting patient-specific management and treatment decision-making. Nevertheless, its efficacy and safety should be further validated through large-scale, multicenter prospective studies.

## Data Availability

The original contributions presented in the study are included in the article/Supplementary material, further inquiries can be directed to the corresponding author/s.
