# Supplementary material for: Safety and efficacy of short-term tirofiban combined with dual antiplatelet therapy after flow diverter placement for intracranial aneurysms: a multicenter retrospective study and nomogram for thromboembolic event prediction
Source: Front Neurol. 2025 Nov 19;16:1689308. doi: 10.3389/fneur.2025.1689308 (PMC12672332; doi:10.3389/fneur.2025.1689308)
Supplement: Supplementary file 1 [file Table_1.DOCX]

| **Table-S1 Angiographic and clinical characteristics  before propensity score matching in treated aneurysms (n=319 patients)** | | | |  |
| --- | --- | --- | --- | --- |
|  |  |  |  |  |
| **Variables** | **DAPT** | **DAPT and tirofiban** | **P-value** |  |
|  | **(N=150)** | **(N=169)** |  |  |
| **Sex** |  |  |  |  |
| Male | 58 (38.7%) | 56 (33.1%) | 0.362 |  |
| Female | 92 (61.3%) | 113 (66.9%) |  |  |
| **Age** |  |  |  |  |
| Mean (SD) | 55.7 (10.5) | 56.4 (11.7) | 0.272 |  |
| **Alcohol** | 19 (12.7%) | 19 (11.2%) | 0.827 |  |
| **Smoking** | 29 (19.3%) | 26 (15.4%) | 0.433 |  |
| **Hypertension** | 71 (47.3%) | 78 (46.2%) | 0.922 |  |
| **Diabetes** | 11 (7.3%) | 18 (10.7%) | 0.404 |  |
| **Symptom** | 98 (65.3%) | 100 (59.2%) | 0.309 |  |
| Dizziness | 44 (29.3%) | 41 (24.3%) | 0.370 |  |
| Headache | 45 (30.0%) | 43 (25.4%) | 0.433 |  |
| Cranial nerve paralysis | 9 (6.0%) | 10 (5.9%) | >0.999 |  |
| **History of stroke** | 0 (0%) | 3 (1.8%) | 0.290 |  |
| **AA** | 10.2 (10.4) | 9.62 (6.59) | 0.623 |  |
| **ADP** | 35.7 (16.3) | 38.3 (14.7) | 0.247 |  |
| **ADR** | 30 (20.0%) | 34 (20.4%) | >0.999 |  |
| **Maximum diameter** |  |  |  |  |
| Mean (SD) | 9.16 (7.17) | 8.42 (6.54) | 0.478 |  |
| **Width** |  |  |  |  |
| Mean (SD) | 7.09 (5.30) | 6.33 (4.74) | 0.177 |  |
| **Neck width** |  |  |  |  |
| Mean (SD) | 6.60 (4.70) | 6.58 (6.63) | 0.157 |  |
| **Diameter of the feeding artery** | |  |  |  |
| Mean (SD) | 3.72 (1.10) | 3.73 (1.51) | 0.496 |  |
| **Aneurysm Location** |  |  |  |  |
| Anterior Circulation | 129(86%) | 140(82.8%) | 0.438 |  |
| Posterior Circulation | 21(14%) | 29(17.2%) |  |  |
| **Admission_mRS** |  |  |  |  |
| 0-2 | 145 (96.6%) | 165 (97.6%) | 0.856 |  |
| ＞2 | 5 (3.4%) | 4 (2.4%) |  |  |
| **Intraoperatively** | | | |  |
| **Thrombosis** | 1 (0.7%) | 0 (0%) | 0.470 |  |
| **Rupture** | 1 (0.7%) | 0 (0%) | 0.470 |  |
| **Branch artery occlusion** | 0 (0%) | 2 (1.2%) | 0.500 |  |
| **Other complications** | 1 (0.7%) | 3 (1.8%) | 0.625 |  |
| **Postoperatively** | | | |  |
| **Ischemic complication** | 5 (3.3%) | 8 (4.7%) | 0.582 |  |
| **Cerebral hemorrhage** | 0 (0%) | 1 (0.6%) | >0.999 |  |
| **Other Complications** | 1 (0.7%) | 6 (3.6%) | 0.125 |  |
| **Death** | 0 (0%) | 1 (0.6%) | >0.999 |  |
| **mRS score（3 Day）** |  |  |  |  |
| 0-2 | 141 (94.0%) | 166 (98.2%) | 0.092 |  |
| ＞2 | 9 (6.0%) | 3 (1.8%) |  |  |
| **mRS score（discharge）** |  |  |  |  |
| 0-2 | 141 (93.9%) | 165 (97.6%) | 0.175 |  |
| ＞2 | 9 (6.1%) | 4 (2.4%) |  |  |
| **Follow-up** | | | |  |
| **Follow up time** |  |  |  |  |
| Mean (SD) | 9.35 (3.65) | 9.79 (3.61) | 0.234 |  |
| **Ischemic complication** | 4 (2.7%) | 13 (7.7%) | 0.081 |  |
| **Hemorrhagic complication** | 4 (2.7%) | 3 (1.8%) | 0.873 |  |
| **Peripheral hemorrhagic** | 4 (2.7%) | 12 (7.1%) | 0.120 |  |
| **mRS score** |  |  |  |  |
| 0-2 | 144 (96.0%) | 166 (98.2%) | 0.390 |  |
| ＞2 | 6 (4.0%) | 3 (1.8%) |  |  |

| **Before PSM Variables** | **DAPT** | **DAPT and tirofiban** | **P-value** |
| --- | --- | --- | --- |
|  | **(N=150)** | **(N=169)** |  |
| **WBC** |  |  |  |
| Mean (SD) | 6.55 (1.91) | 6.59 (2.43) | 0.484 |
| Median [Min, Max] | 6.18 [2.99, 14.3] | 6.03 [1.23, 17.6] |  |
| **LYM** |  |  |  |
| Mean (SD) | 1.96 (0.788) | 1.95 (0.783) | 0.910 |
| Median [Min, Max] | 1.84 [0.190, 5.20] | 1.85 [0.332, 5.50] |  |
| **ANC** |  |  |  |
| Mean (SD) | 4.06 (2.04) | 4.16 (2.35) | 0.684 |
| Median [Min, Max] | 3.88 [0.190, 13.6] | 3.56 [0.170, 16.4] |  |
| **RBC** |  |  |  |
| Mean (SD) | 4.49 (0.547) | 4.42 (0.528) | 0.386 |
| Median [Min, Max] | 4.42 [3.30, 7.79] | 4.42 [2.71, 6.41] |  |
| **PLT** |  |  |  |
| Mean (SD) | 234 (60.5) | 233 (58.7) | 0.615 |
| Median [Min, Max] | 234 [106, 429] | 227 [79.0, 449] |  |
| **HBC** |  |  |  |
| Mean (SD) | 138 (19.8) | 135 (19.3) | 0.234 |
| Median [Min, Max] | 139 [5.60, 231] | 136 [29.1, 214] |  |
| **TG** |  |  |  |
| Mean (SD) | 1.50 (0.923) | 1.39 (0.879) | 0.158 |
| Median [Min, Max] | 1.32 [0.420, 5.89] | 1.17 [0.390, 6.64] |  |
| **TC** |  |  |  |
| Mean (SD) | 4.56 (1.14) | 4.51 (1.05) | 0.772 |
| Median [Min, Max] | 4.49 [1.69, 11.0] | 4.44 [1.80, 8.19] |  |
| **LDL** |  |  |  |
| Mean (SD) | 2.62 (0.997) | 2.56 (0.850) | 0.750 |
| Median [Min, Max] | 2.58 [0.870, 8.29] | 2.52 [0.240, 5.67] |  |
| **HDL** |  |  |  |
| Mean (SD) | 1.37 (0.403) | 1.37 (0.370) | 0.737 |
| Median [Min, Max] | 1.33 [0.640, 3.53] | 1.31 [0.550, 2.71] |  |
| **FIB** |  |  |  |
| Mean (SD) | 2.83 (0.749) | 2.92 (0.700) | 0.270 |
| Median [Min, Max] | 2.88 [0.250, 4.27] | 2.92 [0.420, 5.54] |  |
| **APTT** |  |  |  |
| Mean (SD) | 30.7 (4.17) | 30.5 (4.19) | 0.627 |
| Median [Min, Max] | 30.0 [23.5, 45.8] | 30.2 [3.90, 49.6] |  |

**TableS2** Laboratory tests before propensity score matching in treated aneurysms

| **After PSM Variables** | **DAPT** | **DAPT and tirofiban** | **P-value** |
| --- | --- | --- | --- |
|  | **(N=139)** | **(N=139)** |  |
| **WBC** |  |  |  |
| Mean (SD) | 6.56 (1.96) | 6.62 (2.38) | 0.598 |
| Median [Min, Max] | 6.24 [2.99, 14.3] | 5.95 [2.78, 17.6] |  |
| **LYM** |  |  |  |
| Mean (SD) | 1.90 (0.795) | 1.91 (0.779) | 0.825 |
| Median [Min, Max] | 1.81 [0.190, 5.20] | 1.82 [0.260, 5.50] |  |
| **ANC** |  |  |  |
| Mean (SD) | 3.87 (1.85) | 4.15 (2.53) | 0.705 |
| Median [Min, Max] | 3.78 [0.190, 9.86] | 3.54 [0.470, 16.4] |  |
| **RBC** |  |  |  |
| Mean (SD) | 4.45 (0.480) | 4.45 (0.530) | 0.874 |
| Median [Min, Max] | 4.41 [3.30, 5.60] | 4.43 [2.71, 6.41] |  |
| **PLT** |  |  |  |
| Mean (SD) | 235 (60.4) | 235 (59.1) | 0.699 |
| Median [Min, Max] | 234 [106, 429] | 228 [79.0, 449] |  |
| **HBC** |  |  |  |
| Mean (SD) | 138 (19.9) | 137 (19.5) | 0.834 |
| Median [Min, Max] | 137 [5.60, 231] | 137 [29.1, 214] |  |
| **TG** |  |  |  |
| Mean (SD) | 1.41 (0.712) | 1.37 (0.827) | 0.279 |
| Median [Min, Max] | 1.29 [0.420, 4.20] | 1.17 [0.390, 6.64] |  |
| **TC** |  |  |  |
| Mean (SD) | 4.55 (1.01) | 4.52 (1.08) | 0.712 |
| Median [Min, Max] | 4.49 [1.69, 6.81] | 4.44 [1.80, 8.19] |  |
| **LDL** |  |  |  |
| Mean (SD) | 2.58 (0.890) | 2.60 (0.871) | 0.929 |
| Median [Min, Max] | 2.55 [0.870, 4.43] | 2.60 [0.980, 5.67] |  |
| **HDL** |  |  |  |
| Mean (SD) | 1.39 (0.406) | 1.38 (0.380) | 0.816 |
| Median [Min, Max] | 1.33 [0.640, 3.53] | 1.31 [0.550, 2.71] |  |
| **FIB** |  |  |  |
| Mean (SD) | 2.83 (0.740) | 2.94 (0.702) | 0.570 |
| Median [Min, Max] | 2.89 [0.250, 4.27] | 2.92 [0.420, 5.54] |  |
| **APTT** |  |  |  |
| Mean (SD) | 30.6 (4.20) | 30.7 (4.28) | 0.313 |
| Median [Min, Max] | 30.0 [23.5, 45.8] | 30.2 [3.90, 49.6] |  |

**Table S3** Laboratory tests after propensity score matching in treated aneurysms


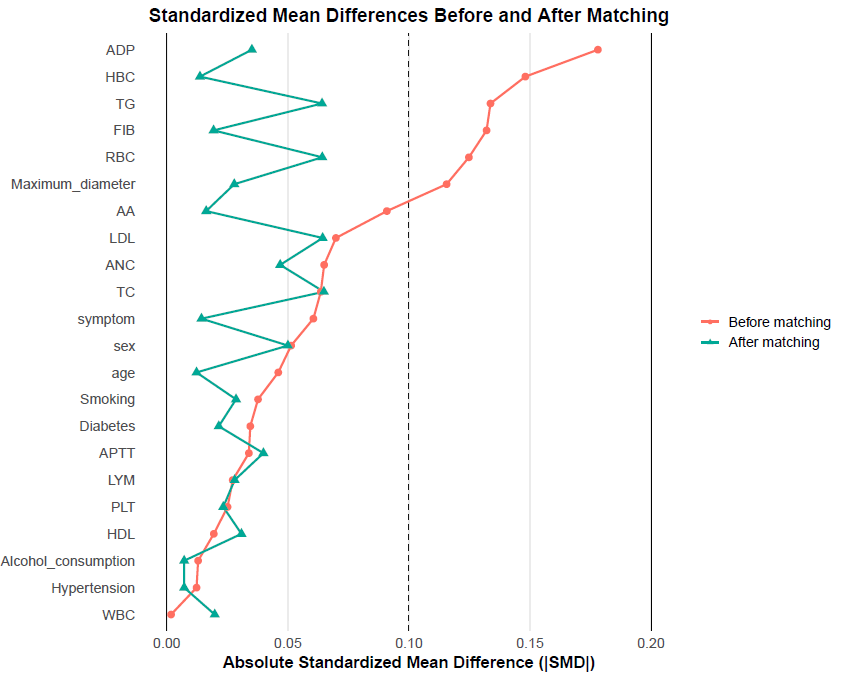


**Figure S1. Standardized Mean Differences Before and After Matching.**

Absolute standardized mean differences (|SMD|) of baseline covariates before (red) and after (green) propensity score matching. After matching, all covariates achieved good balance (|SMD| < 0.1).
